# The Utility of Digital Linear Tomosynthesis Imaging of Total Hip Joint Arthroplasty with Suspicion of Loosening: A Prospective Study in 40 Patients

**DOI:** 10.1155/2013/594631

**Published:** 2013-09-03

**Authors:** Jan H. Göthlin, Mats Geijer

**Affiliations:** ^1^Department of Radiology, Sahlgrenska University Hospital, 431 80 Mölndal, Sweden; ^2^Center for Medical Imaging and Physiology, Skåne University Hospital, Lund University, 221 85 Lund, Sweden

## Abstract

*Aim*. The clinical utility of digital linear tomosynthesis in musculoskeletal applications has been validated in only a few reports. Technical performance and utility in hip prosthesis imaging have been discussed in technical reports, but no clinical evaluation has been reported. The purpose of the current study was to assess the added clinical utility of digital linear tomosynthesis compared to radiography in loosening of total hip joint arthroplasty. *Materials and Methods*. In a prospective study, radiography and digital tomosynthesis were performed in 40 consecutive patients with total hip arthroplasty referred for suspect prosthesis loosening. Tomosynthesis images were compared to anterior-posterior (AP) and cross-table lateral radiographs regarding demarcation and extent of demineralization and osteolysis. Further noted were skeletal fractures, cement fractures, fragmentation, and artifacts interfering with the diagnosis. *Results*. Tomosynthesis was superior to radiography with sharper delineation of demineralization and osteolysis in the AP projection. A limitation was the inability to generate lateral tomosynthesis images, with inferior assessment of the area anterior and posterior to the acetabular cup compared to cross-table radiographs. Artifacts interfering with diagnosis were found in one hip. *Conclusion*. Tomosynthesis improved evaluation of total hip arthroplasty in the AP projection but was limited by the lack of lateral projections.

## 1. Introduction

Imaging of hip arthroplasty is an important tool to evaluate the postoperative placement of components [1] and later to evaluate possible complications [2]. This is commonly done using radiography with an anterior-posterior (AP) pelvis and hip acquisition and a cross-table lateral acquisition. In addition computed tomography (CT) may be used both for preoperative planning and postoperative followup, while magnetic resonance imaging (MRI), nuclear medicine studies, and ultrasound examinations mostly are used for evaluation of postoperative complications.

For radiographic evaluation of total hip arthroplasty the Gruen scale is used to assess cement fractures and radiolucent zones along the cement-bone or cement-prosthesis interface [3]. The proximal femur on the AP radiograph is divided into seven zones, each evaluated individually, to which later seven lateral zones were added. The Barrack grading may be used to describe the quality of the cement mantle surrounding the femoral stem [4]. The acetabular cup may be evaluated according to Hodgkinson et al. [5]. In a recent study evaluating these indices for prosthesis complications [6] using digital images and a picture archiving and communication system (PACS) all three indices were found to have limited inter- and intrareader agreement, making the authors question their reliability in evaluating loosening of total hip arthroplasties. The results from that study were similar to a previous study using conventional radiographs [7], where intraobserver agreements generally were moderate, but interobserver agreement was poor, sometimes even less than a chance, making the authors question the reliability of comparisons between studies from different centers.

CT (and MRI) are better than radiography in evaluating osteolysis due to their tomographic nature [8]. Both methods, however, have difficulties in evaluating the prosthesis-bone or prosthesis-cement interface due to metallic artifacts. Even though artifact-reducing image reconstruction algorithms have been created, the problem still remains. Another problem for CT is the higher effective dose than that from radiography. CT is currently not used for systematic prosthesis evaluation clinically or in clinical studies but is used for preoperative planning in difficult cases with better delineation of osteolysis than radiography [9, 10], for evaluation of suspected osteolysis or granulomas in painful hips, and for evaluation of iliopsoas muscle and tendon complications.

Radiography may be refined with tomography which was early introduced in conventional radiology [11]. The technique has been further refined in digital radiography as tomosynthesis [12], referring to the ability to retroactively create an infinite number of arbitrary tomograms. Tomosynthesis improves upon conventional geometric tomography in that it allows an arbitrary number of in-focus planes to be generated retrospectively from a sequence of projection radiographs that are acquired during a single sweep of the X-ray tube using reconstruction techniques such as filtered back projection [13]. Modern linear tomosynthesis is performed in a conventional radiography suite, where dedicated software allows the moving X-ray tube to perform a 10-second linear sweep directed towards the stationary detector, during which time, about 60 low-dose exposures are obtained [14]. From these exposures up to 60 sectional images may be reconstructed in the plane of the detector at an arbitrary thickness from 1 to 10 mm. These reconstructions are later evaluated in stack mode in the picture archiving and communication system (PACS). 

The potential usefulness of digital linear tomosynthesis for evaluation of hip arthroplasty has been demonstrated in one patient by Gomi and Hirano [15].

Several articles have been published reporting on the utility of tomosynthesis in chest radiology [16–19] where it in certain applications may be used as a low-dose alternative to chest CT. The principle has been used in mammography for more than a decade [20]. Technical developments have now led to the introduction of the technique also for abdominal [21, 22] and musculoskeletal imaging [23–26]. Perusal of the literature has not revealed any prospective study of the clinical utility of tomosynthesis of hip prosthesis with clinical suspicion of loosening.

The aim of the current study was to evaluate the added utility of digital linear tomosynthesis imaging to plain radiography in suspect of loosening of hip arthroplasties.

## 2. Material and Methods

### 2.1. Patients

The current prospective study was approved by the local ethics committee, and written informed consent was obtained from all participants. The study population consisted of 40 consecutive patients (18 women, mean age 68 years, range 40–81 years and 22 men, mean age 68 years, range 46–87 years) referred for radiography and digital linear tomosynthesis with suspicion of prosthesis loosening with increasing pain or instability or for preoperative assessment. The implants were of different models from seven different manufacturers. Thirty-three patients had cemented femoral stems and cemented acetabular cups. Five patients had an uncemented femoral stem with a cemented acetabular cup. Two patients had uncemented femoral stems and acetabular cups. Thirty-one implants were cobalt-chrome, two stainless steel, and seven titanium.

### 2.2. Image Acquisition

Radiography was performed using a Definium 8000 radiography system from GE Healthcare (Chalfont St Giles, UK) with cross-table lateral images acquired with Fuji storage phosphor image plates (Fuji, Tokyo, Japan). Pixel spacing was 0.2 × 0.2 mm for the GE system, 0.15 × 0.15 mm for the Fuji plates. Focus-detector distance was 100 cm for the AP images. For AP tomosynthesis 75 kV was used, for AP radiography 70 kV. Cross-table lateral images were acquired with a setting of 90 kV and 80 mAs.

Tomosynthesis was performed with a commercially available product (Definium 8000; GE Healthcare, Chalfont St Giles, UK; VolumeRAD; GE Healthcare), described in detail in several reports [14, 16, 27, 28]. Briefly, about 60 low-dose exposures are acquired by a moving X-ray tube directed towards a stationary digital amorphous silicon flat-panel detector, from tube angles from −15° to +15°. They are used to reconstruct up to 60 coronal sectional images by using filtered back projection (FBP), commonly simplified in descriptions as a shift-and-add technique and various deblurring algorithms. The scan time is approximately 10 s. In tomosynthesis the multiple tomographic sections have an appearance similar to radiographs and conventional tomograms, with sharply depicted structures in the focal plane of each section and blurred structures outside the section.

In the radiography system used for the current study, an AP hip radiograph serving as a scout image is automatically included in the tomosynthesis scan. For the purpose of this analysis, hip radiography consisting of an AP radiograph (the tomosynthesis scout image) and a cross-table lateral radiograph were compared to a tomosynthesis scan consisting of AP reconstructions of 2 mm nominal thickness without overlap, a setting recommended by the vendor. The current study compares only the sectional images from tomosynthesis with the conventional AP and cross-table lateral radiographs. The tomosynthesis sections and the AP hip radiograph covered the same anatomical region.

Measurements of dose were retrieved from the automatic dose registration system. In that system, the dose-area product value (DAP value) is stored. The DAP value was calculated by the X-ray system from the selected collimation, energy, tube load, and copper filtration. Separate registrations for the tomosynthesis acquisition and the combined AP acquisitions of pelvic and AP hip radiographs were available. The cross-table lateral hip radiograph had at the time of the study not been included in the automatic dose registrations.

The effective dose (*E*) is calculated from the registered DAP values by using the formula:

(1) $$\begin{array}{c} E=E_{\text{DAP}}*\text{DAP (mSv)}, \end{array}$$

where DAP (Gycm^2^) is the dose-area product as calculated by the X-ray equipment during the exposure and *E*_DAP_ is the conversion factor in mSv/Gycm^2^. The conversion factor, 0.29 mSv/Gycm^2^, was obtained from the Swedish Radiation Protection Authority [29].

### 2.3. Image Assessment

The imaging studies were evaluated with the normal clinical PACS at our institution (Sectra IDS 7, Sectra, Linköping, Sweden) on Barco color medical-grade diagnostic monitors with a pixel resolution of 3280 × 2048 (Barco Inc., Duluth, GA, USA). Free use of all PACS tools was allowed, with unlimited viewing time. Both observers were familiar with tomosynthesis imaging for three years and hip prosthesis evaluation for more than 20 years from their normal clinical work, and both were musculoskeletal radiologists with more than 20 years' of experience.

The image studies were assessed by both authors in consensus. The tomosynthesis scans were scored as superior, equal, or inferior to standard hip AP radiographs and cross-table lateral projections regarding demarcation and extent of demineralization around the cup and stem, respectively, as well as regarding differentiation between demineralization and osteolysis in a side-by-side comparison. Assessment was also made of the prevalence and extent of skeletal fractures, cement fractures, and cement fragmentation. Artifacts interfering with the diagnosis were noted. The possible improved diagnosis of one modality over the other was appraised. The findings were summarized in an assessment of the need for a cross-table lateral radiograph in determining the total extent of demineralization and osteolysis.

Clinical correlation was not obtained as the purpose of the study was to evaluate the added utility of tomosynthesis in evaluating hip arthroplasty radiography.

## 3. Results

**Figure 1:**
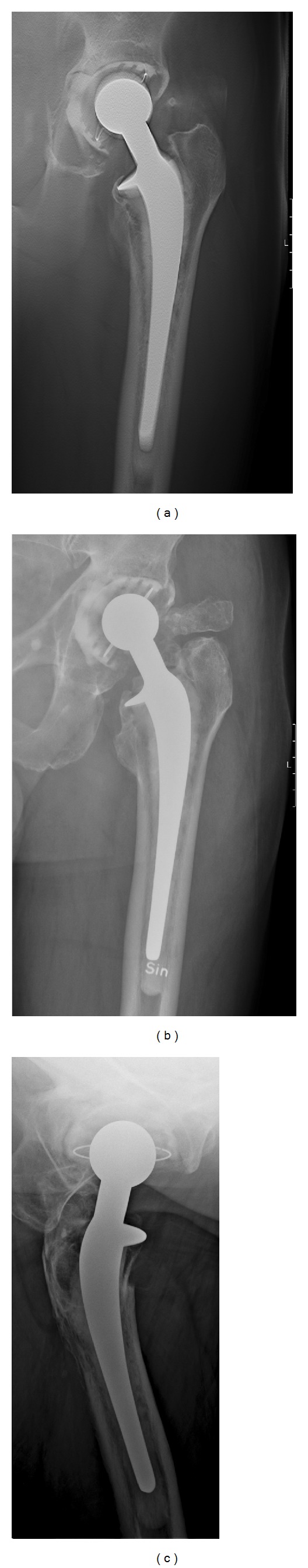
A 75-year-old man with hip pain and suspected complications of a total hip arthroplasty. (a) A tomosynthesis section through the middle of the hip prosthesis, showing an improved delineation of the osteolysis around the cemented cup, especially the large erosion medial to the cup (arrows). (b) The AP radiograph does not display the osteolysis to full advantage. (c) The cross-table lateral radiograph shows the osteolysis anterior and posterior to the cup (arrowheads), which cannot be appreciated neither with tomosynthesis nor on the AP radiograph.

Tomosynthesis and radiography scored as equal in differentiating between demineralization and osteolysis around the cup and stem in 29 of 40 cases (72%, Table 1). Tomosynthesis was scored as superior in the other 11 cases (28%). In more than half of the cases tomosynthesis was scored as superior to radiography in the sharpness of demarcation of both demineralization and osteolysis. Tomosynthesis proved superior to radiography in most cases in sharper demarcation of the extent of demineralization and osteolysis around the hip prostheses (Figure 1, Table 1) compared to the conventional AP projection. The sagittal extent was better assessed with the cross-table lateral projection which in 24 cases was necessary to determine the total extent of osteolysis. 

The material contained one femoral stem fracture only, which could be somewhat better visualized with tomosynthesis. Around one cup and one stem cement fractures and fragmentation could be somewhat better seen with tomosynthesis. However, the diagnoses were in these cases not altered. The sagittal extent and differentiation of demineralization and osteolysis were in most of the cases much better assessed on cross-table lateral radiography projections than with AP tomosynthesis. Disturbing metal artifacts interfering with image evaluation could be seen around one stem with tomosynthesis.

No significant differences could be found between the results for cemented and noncemented prosthesis components.

The mean effective dose from the tomosynthesis scan was 0.9 mSv (range 0.1–5.2 mSv). Mean effective dose for AP pelvis and hip radiography combined was 1.1 mSv.

## 4. Discussion

Modern tomosynthesis using a large digital detector has been feasible for several years but has still not been widely evaluated in the musculoskeletal field. There are few studies, which generally report added clinical utility compared to radiography [23] but less utility than with CT [25, 26]. Several reports [15], abstracts, and posters [27, 28] have discussed the possibilities for improving on hip prosthesis radiography with tomosynthesis in phantom studies or in case reports, but no clinical material has previously been reported. 

In the current study on a clinical prospective material, mainly demarcation and extent of demineralization and osteolysis were better seen with tomosynthesis than with radiography in the AP projection (Figure 1). In the thin tomosynthesis sections without interference from overlying soft tissues the demarcation between osteolysis and surrounding bone was more distinct than in the projection radiographs. Similarly, the differentiation between periprosthetic demineralization, which to a large extent is an evaluation of tissue density, and osteolysis was easier using the thin tomosynthesis sections. In most cases, however, the changes were easily detectable also with radiography, and tomosynthesis mainly added a more detailed evaluation. In general, tomosynthesis thus increased diagnostic confidence but did not change diagnosis from radiography, without added clinical utility from tomosynthesis compared to radiography.

The improved differentiation and visualization of demarcation and extent of demineralization and osteolysis with tomosynthesis in the current study apply to the AP projection only. A limitation of tomosynthesis, as with only utilizing an AP radiograph, is that changes localized anterior or posterior to the prosthesis cannot be adequately evaluated and will be better visualized with the cross-table lateral radiographic view. Linear tomosynthesis cannot generate reconstructions in other imaging planes than that of the detector, and sagittal reconstructions cannot be made unless the patient is rotated 90 degrees. It is possible to rotate the hip, as shown by Gazaille et al. in a report on hip arthrography with tomosynthesis [24], which would display the femoral stem from lateral, but the acetabular cup would still be displayed in mainly the AP projection. To our knowledge, this problem has not been solved technically by any vendor. Also, the area cranioanterior and cranioposterior to the acetabular cup suffers from shadowing artifacts when using tomosynthesis and is also best evaluated on the cross-table lateral projection. A cross-table lateral radiograph is thus required to evaluate the entire prosthesis, with tomosynthesis as well as in conventional radiography. Thus, a tomosynthesis scan only cannot replace the conventional radiograph, but may serve as an adjunct providing a more detailed information in the AP projection.

In the current study comparing tomosynthesis to conventional radiography, there were no artifacts from the prosthesis hardware interfering with diagnosis except in one case, where a minimal osteolysis close to a femoral stem was concealed by the metal artifacts. In general, ghosting artifacts with tomosynthesis have been considered much less problematic than with computed tomography (CT) in the literature [15, 30]. Metallic artifacts from tomosynthesis are different from metallic artifacts from CT, in that they are a combination of superimposition of metallic structures and ghosting, whereas in CT, they are a result of photon starvation resulting in ghosting and streak artifacts. Generally, the metallic artifacts as such are not very troublesome in hip prosthesis imaging, since they extend in the scan direction and only influence the area around the tip of the stem and the top of the acetabular cup and only on the sections anterior and posterior to the vertex of the cup or the stem (Figure 1).

In the comparison of methods, direct measurements of widths of resorption zones or subsidence were not used due to difficulties in assigning valid measuring points from projection radiographs on sectional images and also due to the purpose of the study to assess the possible added value of tomosynthesis to conventional hip prosthesis radiography, not its replacement. An observer agreement evaluation was not performed in the current study. Previous studies on musculoskeletal tomosynthesis [23, 26] and chest tomosynthesis [19] have reported higher agreement figures for tomosynthesis than for radiography but less than for CT [26].

The general advantages of tomosynthesis in the current study were that it was quick and could be performed directly after radiography without rescheduling of the patient, using the same radiographic equipment in the same radiography suite as for conventional radiography, and adding only about one minute to the total examination time. The organ dose as well as the effective dose of tomosynthesis has been evaluated with an anthropomorphic phantom [31]. The effective dose from hip radiography in the AP projection (not including the cross-table lateral view) was reported as 0.088 mSv while the dose from a tomosynthesis scan was reported as 0.82 mSv. In the current study, the mean effective dose was similar, 0.88 mSv, with a wide range of measurements. The variation among measurement can be explained by differences in patient weight and factors such as collimated area and image centering. Båth et al. [14] evaluated the effective dose from tomosynthesis in chest radiography using Monte Carlo simulations and reported the average effective dose from a posterior-anterior radiograph as 0.014 mSv, while the average tomosynthesis scan dose was reported as 0.122 mSv. In both cases the tomosynthesis dose was about nine times the radiography dose. However, in both instances the cross-table and lateral views, respectively, contribute to most of the radiography dose; in the case of chest radiography the lateral radiograph gave an average effective dose of 0.039 mSv [14]. The added effective dose from tomosynthesis to the complete radiography study is thus about 2–5 times that of radiography [14, 31] and is low compared to CT. The disadvantage of tomosynthesis in the current study is the limited additional clinical utility compared to radiography in evaluating loosening of total hip arthroplasty. There are also additional images to review with longer reporting time, and the extra examination is associated with extra costs.

The limitations of the current study are mainly that the material is small and does not permit differentiation of the results between cemented and noncemented stems. However, the purpose of the study was to evaluate the added diagnostic utility of tomosynthesis in hip arthroplasty in general not to evaluate different prosthesis types. Another limitation is that the images were reviewed in consensus and in a side-by-side comparison. However, it is known that observer agreements for radiographic evaluation of total hip arthroplasty using various scoring systems are only fair to good between observers of different experience [6, 7], and previous studies on tomosynthesis [19, 23, 26] have reported higher agreement figures for tomosynthesis than for radiography. Another limitation is the fact that there was no imaging or clinical reference in the study. Reference imaging with CT [9, 32, 33] or arthrography [8, 34] was not performed in the study. Both modalities would have resulted in a higher dose to the patients. It is possible to perform modern musculoskeletal CT at sub-mSv doses, but the possibility of using such low doses with metallic implants such as hip arthroplasties has yet not been shown. In a study on preoperative assessment on native hips before hip arthroplasty, the mean effective dose of hip CT was reported as 4.0 mSv [35]. 

In conclusion, tomosynthesis of hip arthroplasty gave an improved visualization of demineralization and osteolysis extent and demarcation in the current study compared to radiography, which may make imaging studies easier to read and evaluate. Tomosynthesis may thus improve on radiographic evaluation of prosthesis loosening which may be important, considering the low to moderate observer agreement figures reported in previous studies [6, 7]. The cross-table lateral hip radiograph cannot, however, be replaced by tomosynthesis, and it is yet not possible to give clear recommendations on the use of tomosynthesis in hip prosthesis evaluation. Further studies on larger materials are needed.

## Figures and Tables

**Table 1:** The results of the AP radiography projection compared to the AP tomosynthesis scan in evaluation of demineralization and osteolysis around total hip arthroplasties. The cross-table lateral projections were not included in the comparison.

Demineralization and osteolysis	Radiography inferior	Radiography and tomosynthesis equal	Radiography superior
Differentiation	11 (28%)	29 (72%)	0 (0%)
Demarcation	23 (58%)	16 (40%)	1 (2%)
Extent in the coronal plane	33 (82%)	6 (15%)	1 (2%)
